# Drug-Induced Sexual Dysfunction in Individuals with Epilepsy: Beyond Antiepileptic Compounds

**DOI:** 10.3390/medicines9030023

**Published:** 2022-03-14

**Authors:** Rocco Salvatore Calabrò, Antonio Cerasa

**Affiliations:** 1 IRCCS Centro Neurolesi “Bonino-Pulejo”, 98123 Messina, Italy; 2 Institute for Biomedical Research and Innovation (IRIB), National Research Council of Italy (CNR), 98100 Messina, Italy; antonio.cerasa@irib.cnr.it; 3 S’Anna Institute, 88900 Crotone, Italy

Sexual dysfunction (SD) is a multifactorial problem, involving neurological, iatrogenic, endocrine, psychiatric, and psychosocial factors, and affects the quality of life of both male and female individuals with epilepsy [1,2]. Among the iatrogenic factors, both older and newer antiepileptic drugs are known to potentially determine SD [3]. In particular, liver enzyme-inducing antiepileptic drugs (AEDs), such as phenobarbital, phenytoin, and carbamazepine, can cause SD by decreasing bioactive testosterone, accelerating sexual hormone metabolism, and stimulating the production of the hormone binding globulin, whereas new AEDs are thought to cause SD through still poorly understood mechanisms, mainly involving cerebral neurotransmission with an imbalance in the serotonin/dopamine ratio [3]. Valproate has a detrimental effect on female fertility by causing polycystic ovary syndrome [1].

It is well-known that patients with epilepsy have a higher prevalence of psychiatric disorders, including anxiety, depression, and interictal dysphoria [4]. This is why most of these disorders are also treated with psychotropic drugs, despite the believed risk of drug-induced seizure exacerbation. The evidence shows that the majority of psychotropic medications are not proconvulsant when used at therapeutic doses, with the exception of bupropion and certain antipsychotic drugs such as clozapine [5]. However, the effectiveness of antidepressant drugs for depression in epilepsy is unknown [6]; thus, it should be mandatory to take into consideration the potential side-effects—including sexual ones—before prescribing such drugs. 

Antidepressants are most likely to cause SD [7]. This is possibly due to an imbalance in the dopamine/serotonin ratio, considering that dopamine exerts a positive effect on sexuality, whereas most serotonin receptors negatively affect sexual function [8]. In fact, selective serotonin reuptake inhibitors (SSRIs) have previously been associated with adverse effects on all three phases of sexual function, although their most prominent side-effects include delayed ejaculation, reduced lubrication and anorgasmia [9]. The effects of SSRIs on arousal and orgasm may be mediated by the stimulation of the 5HT2 receptors of the serotonergic pathway involving the medullary raphe nuclei and the spinal cord, whereas the decrease in libido may be due to reduced dopaminergic activity in the mesolimbic system [8]. Notably, there is some evidence that noradrenergic effects may mitigate serotonin’s influence on sexual function. Indeed, reboxetine, a norepinephrine transporter inhibitor, seems to have a lower SD prevalence than other antidepressants [10]. Moreover, iatrogenic SD due to duloxetine and venlafaxine, two common serotonin noradrenaline reuptake inhibitors (SNRIs), has proven to cause less SD than other SSRIs [10]. The newer SNRI vortioxetine seems not to cause any SD, thanks to its multimodal modulation of the serotonin receptors [11]. Owing to the dual inhibition of norepinephrine and dopamine reuptake, bupropion is devoid of any direct effects on the serotonin system and has the potential to positively affect arousal and desire [10]. Patients should be counseled about the potential for antidepressant-induced changes in their sexual function and told that such changes can be managed. Possible alternatives include waiting for tolerance to appear, decreasing the dosage, giving drug holidays, adding an additional drug, and switching to an alternative antidepressant less likely to cause SD. In most cases, sildenafil could be of help to improve both erectile function and ejaculation [12], but care should be taken in patients with epilepsy because of the drug’s potential to induce seizures if not used adequately [13]. 

Nonetheless, the different approaches to managing iatrogenic SD could lead to an exacerbation of the depressive symptomatology. There is a bidirectional relationship between sexual function and depression: the presence of either one of these conditions may trigger or exacerbate the other, and the treatment of one condition may improve the other [14]. Indeed, the two syndromes have dysfunctions of common neurotransmitter systems, and the drug used to treat depression may worsen and/or improve SD (e.g., paroxetine is effective in treating premature ejaculation because they cause delayed ejaculation). It is noteworthy that SD may persist for a long time after the drug has been withdrawn. The so-called post-SSRI syndrome is considered a new challenge because, to date, no specific treatment exists and diagnostic criteria are far from being accepted by the academic world [15]. All these issues should be addressed when counselling and treating patients with epilepsy and depressive symptoms. 

Psychotic symptoms in epilepsy can arise in a number of different clinical scenarios, including peri-ictal symptoms, chronic interictal psychosis, comorbid schizophrenia and disorders related to the so-called forced normalization phenomenon. There are still limited data on the best treatment for psychotic disorders in epilepsy, although second-generation antipsychotics, especially risperidone, represent a reasonable first-line option due to their low propensity for drug–drug interactions and low risk of seizures [16]. Antipsychotics may affect sexuality in different ways [7]. Decreased libido is very common with older antipsychotic drugs, such as chlorpromazine and haloperidol, because they are potent dopamine blockers increasing prolactin levels. Indeed, it has been reported that up to 60% of patients taking such medications have experienced SD [7,17]. Among the newest atypical antipsychotic drugs, risperidone is most likely to cause elevations in prolactin levels as well as hyperprolactinemia-related symptoms, such as gynecomastia, erectile dysfunction, reduced lubrication and decreased libido. Moreover, risperidone has also been associated with ejaculatory dysfunction, such as retrograde ejaculation, as well as reduced lubrication. On the contrary, hyperprolactinemia has rarely been associated with quetiapine, ziprasidone, aripiprazole (which appears to be a drug with fewer SD in males), or clozapine [17,18]. Notably, atypical antipsychotics may cause additional adverse effects on arousal and orgasm function because of their negative activity on the serotonergic pathway [18]. 

Benzodiazepines are known to increase the inhibitory process of the central nervous system primarily through aminobutyric acid, the main inhibitory neurotransmitter [8]. Although the overall risk of SD with benzodiazepines is relatively low, high-dose therapy has been associated with an increased incidence of SD [19]. Thus, attention should be paid when prescribing these drugs, including clobazam, which is often used to treat both seizures and psychogenic non-epileptic seizures in patients with epilepsy.

Finally, older patients with epilepsy may also be affected by other comorbidities, including hypertension, diabetes, and dyslipidemia. Indeed, stroke is the most common cause of seizures and epilepsy at old age [20]. Although cardiovascular risk factors are known to cause SD, most of the sexual problems of concern for these patients may be related to the drug used to treat them, including antihypertensives. These drugs are known to cause SD, including erectile dysfunction, ejaculation disorders and reduced sexual desire. Angiotensin II receptor antagonists exert the most severe effect on sexual function, especially when combined with diuretics, which impair sexuality through different and still not well-known mechanisms, including lowering of blood volume and flow and decreasing electrolytes [21]. Beta blockers, except nebivolol, determine SD through their action on the adrenergic system, whose balance with the cholinergic pathway is fundamental for a vasogenic erection to exist [8]. Calcium channel blockers and ACE inhibitors appear to have the best profiles, leading to fewer sexual side-effects than other antihypertensive drugs [21].

In conclusion, because sexual function is an integral and fundamental part of the quality of life of individuals with epilepsy, clinicians should always investigate drug-induced SD. Unfortunately, no guidelines on how managing iatrogenic SD in patients with epilepsy exist and, therefore, research in this field should be fostered to further improve quality of life in this patient population. 
